# What Aspects of Phenotype Determine Risk for Sudden Cardiac Death in Pediatric Hypertrophic Cardiomyopathy?

**DOI:** 10.3390/jcdd9050124

**Published:** 2022-04-21

**Authors:** Ingegerd Östman-Smith

**Affiliations:** 1 Department of Pediatrics, Institute of Clinical Sciences, Sahlgrenska Academy, University of Gothenburg, 416 85 Gothenburg, Sweden; ingegerd.ostman-smith@pediat.gu.se; Tel.: +46-31-3434512; Fax: +46-31-190484; 2 Queen Silvia Children’s Hospital, Sahlgrenska University Hospitals, 416 85 Gothenburg, Sweden

**Keywords:** hypertrophic cardiomyopathy, risk factors, sudden cardiac death

## Abstract

Sudden cardiac death due to hypertrophic cardiomyopathy (HCM), is the most common autopsy-proven cause of unexpected medical death in children after infancy. This mode of death is preventable by implantation of an internal cardiac defibrillator (ICD), a procedure that has considerable morbidity in childhood patients, and even mortality. Since HCM is an inheritable disease (usually autosomal dominant, occasionally recessive), family screening may identify subjects at risk. This review summarizes published studies carried out to identify which phenotypic markers are important risk factors in childhood patients with HCM and reviews the performance of existing risk-stratification algorithms (HCM Risk-Kids, PRIMaCY) against those of single phenotypic markers. A significant proportion of HCM-patients diagnosed in childhood are associated with RASopathies such as Noonan syndrome, but a knowledge gap exists over risk stratification in this patient group. In conclusion, pediatric risk-stratification algorithms for sudden cardiac death perform better in children than adult HCM risk-stratification strategies. However, current multivariable algorithms overestimate risk substantially without having high sensitivity, and remain ‘a work in progress’. To include additional phenotypic parameters that can be reproducibly measured such as ECG-markers, e.g., ECG risk score (which has high sensitivity and negative predictive value), tissue Doppler diastolic function measurements, and quantification of myocardial scarring on cardiac magnetic resonance imaging, has the potential to improve risk-stratification algorithms. Until that work has been achieved, these are three factors that the clinician can combine with the current algorithm-calculated per cent risk, in order better to assess risk.

## 1. Introduction

From the earliest description of hypertrophic cardiomyopathy (HCM), the disease was always defined by its phenotypic expression, and the European Society of Cardiology (ESC) 2014 Guidelines define it: “Hypertrophic cardiomyopathy (HCM) is defined by the presence of increased left ventricular (LV) wall thickness that is not solely explained by abnormal loading conditions. This definition applies to children and adults and makes no a priori assumptions about aetiology or myocardial pathology” [1]. In adulthood, the diagnostic threshold is a left ventricular (LV) wall thickness ≥ 15 mm in one or more myocardial segments, or ≥13 mm if close family history or other supportive clinical features including ECG abnormalities are present [1]. This definition introduces a sex bias in diagnosis, as normal adult female wall thickness is about 1 mm less than it is in males [2,3]. Thus the diagnostic cut-off is higher above normal for females than for males. In childhood, the definition is an LV wall thickness Z-score > 2, where a Z-score is defined as the number of standard deviations from the population mean in normal subjects [1], with normal or increased systolic function and a non-dilated cavity. Most Z-scores are related to body surface area, which removes the issue of a diagnostic bias against females in childhood. 

Using the above definitions, the prevalence of HCM in adults has been found to be 1:500 in a number of ethnically distinct populations [4,5], and very considerably lower in childhood, being 2.9 per 100,000 age-specific population in a national cohort, and with a yearly incidence of new cases between 0.22 and 0.24 per 100,000 [6,7]. However, these studies were performed before widespread cascade screening was advocated, so might represent some underestimation of childhood prevalence as they are based on children presenting with symptoms or physical signs in 75% of cases, with only 25% diagnosed after family screening [6]. That a diagnosis of HCM in childhood denotes a worse survival than a diagnosis in adulthood was recognized already in 1981 [8], and apart from in infancy, where heart failure deaths predominate, the most common cause of death is sudden unexpected arrhythmia [9,10,11]. Sudden cardiac death due to HCM is in fact the most common autopsy-proven cause of unexpected medical death in children after infancy in both Sweden and Japan [12,13], and also the most common cause of unexpected death in young athletes [14]. This is a mode of death preventable by the implantation of an internal cardiac defibrillator, a procedure which however has considerable morbidity in childhood patients, and even mortality. Trying to identify the individual patient at increased risk is therefore clinically very important and is the subject of this review.

## 2. At What Age Is the Risk Highest?

**Figure 1:**
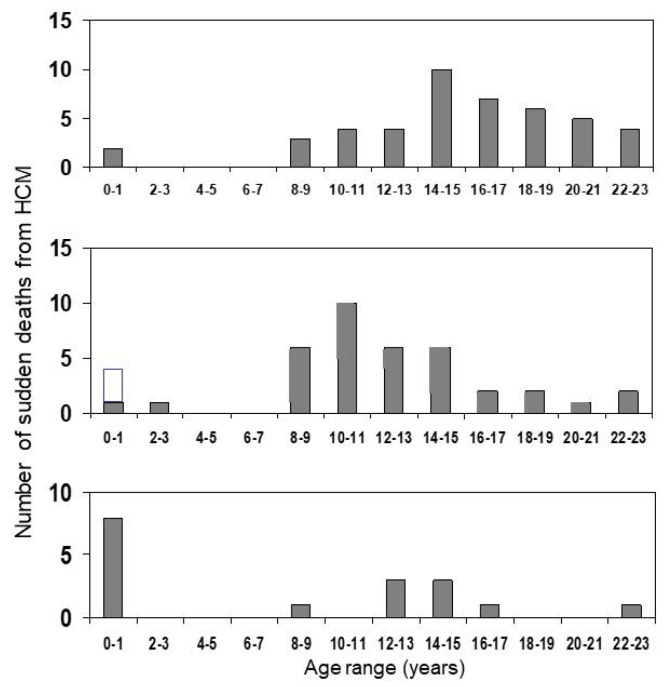
Shows a bar diagram illustrating number of SCDs at different ages in three different studies including childhood-HCM [12,15,16,17].The open part of the first bar in the middle section of the figure illustrates infant cases diagnosed at autopsy where a metabolic cause has not been excluded.

There is a widespread habit of researchers studying the incidence of sudden cardiac death (SCD) to try to quantify the risk in terms of per cent annual mortality. This may seem a convenient short-hand for comparing SCD-mortality in different studies, but is scientifically incorrect, as the rate of SCD in different age bands varies significantly, being very low between 1–7 years of age, and higher in the 8–16 year age range; the mortality in the 8–16 year age range is also significantly higher when compared with the 17–30 year age range [12,15]. The degree of age-dependence of the risk is illustrated in Figure 1**,** which shows the number of SCDs occurring at different ages in three different studies. Viewing these graphs, one also needs to remember that the prevalence of diagnosed HCM cases is higher in later teenage years as compared to young children, with the median age at diagnosis ranging between 6 and 13 years of age in most studies of childhood-HCM (see Table 1 below).

In a Swedish national cohort study, including cases first presenting with SCD diagnosed at autopsy, the population-based risk of SCD was assessed by audited death certificates between 1997–2002. The annual mortality rate was 0.055 per 100,000 age-specific population in 17–30 year olds, i.e., young adults, versus 0.112 per 100,000 age-specific population in 8–16 year olds, and very low in the 0–7 year group; in the 0–7 year group, the majority of the few HCM deaths were related to heart failure [12]. This study is the only study to assess risk against age-specific population size. Studies in the multi-national PRIMaCY-cohort also confirm that the risk of SCD is extremely low below five years of age [18]. Thus it is clear that the time of adrenarche and the pubertal growth spurt is a period of enhanced risk for SCD. This age-dependent variation in SCD-risk means that mortality rates ought only to be compared between studies with similar age composition of cases. One study that attempted to quantify the risk in age bands, i.e., relating the number of SCDs to the number of cases already diagnosed at that particular age band, arrived at an estimate of the highest annual risk of 7.2% between 9–12 years of age, as compared to an annual risk of 1.7% between 16–19 years of age, and a risk close to zero between 1–7 y [12]. This calculation included cases presenting with sudden death [12]. In a Swedish national cohort, 29/39 SCDs occurred in the 8–19 year old age range, and there was only a single SCD below 8 years of age [19]. The median age at SCD was 15.4 [IQR 11.8–22.0] [19], which might appear paradoxical until you realize that the prevalence of overt HCM is substantially higher in 16 year olds than it is in 9 year olds. Confirming that the risk of SCD is highest in childhood cases diagnosed at a young age coming from an Italian cohort in which children diagnosed with HCM at an age of ≤12 years had a higher risk of SCD-events than children diagnosed after the age of 12 years [20].

As illustrated in Table 1, the actual SCD-mortality reported also depends on whether the estimate includes cases that presented with SCD and were only diagnosed at autopsy, or only includes cases that were under cardiac follow-up before death [15]. Studies from earlier eras, which probably contained a higher proportion of untreated cases, as well as a higher proportion of cases that presented with symptoms or physical signs due to outflow tract obstruction, also tend to report higher SCD-mortality than later studies. More recent studies attempting to recruit only non-syndromal HCM cases probably contain a higher proportion of asymptomatic patients diagnosed by family screening, as well as patients with a more active medical management, and have reported lower rates of SCD and arrhythmia end-points such as appropriate ICD-discharge. To compare the different studies we have to resort to annual mortality, as recent authors do not report data on age-specific prevalence, but average all the ages from diagnosis, thereby including also the low-risk 0–7 year olds in the calculations. 

**Table 1:** Comparison between rates of malignant arrhythmia events in different studies.

Era	Age D (y)	Setting (n)	FU (y)	Patient yrs	Events (Type)	“Annual” Event rate (%)	Incl. Comm. SCD?	Exclusions	Reference
1962–1980	9	SC, TC (37)	9	333	18 (SCD)	4.8	No	FH of SCD	McKenna et al., 1984 [21]
1958–1997	5.0	SC, TC (99)	4.8	475	12 (SCD)	2.5	No	None	Yetman et al., 1998 [22]
1968–1998	6.3	MC, RC (66)	12.0	789	10 (SCD)	Total:1.3 HDBB = 0 NST: ns-HCM = 2.3 RAS = 2.3	No	None	Östman-Smith et al., 1999 [23]
1970–2003	5.7	MC, RC (128)	ns:10.9 RAS:12.0	Ns = 948 RAS = 492	Ns = 12 RAS = 4 (SCD)	ns-HCM = 1.3 RAS = 0.8	No	None	Östman-Smith et al., 2005 [9]
1972–2004	4.6	MC, Nat Cohort (150)	7.0	1050	39 (under FU = 27) (LAE)	Total incl cSCD: 3.7 FU-grp = 2.0	**Yes**	None	Östman-Smith et al., 2008 [12]
1985–2006	10.6	SC, TC (96)	6.4	614	3 (SCD)	0.5	No	None	Decker et al., 2009 [24]
1980–2001	?(14.4 at EP-study)	SC, TC (131)	6.4	838	22 (MACE)	2.6	No	Prev MACE	Moak et al., 2011 [25]
1993–2014	14.1	SC, TC (112)	6.5	728	13 (LAE)	1.8	No	None, phenocopies included	Ziolkowska et al., 2015 [26]
1987–1996	0.45	MC, nat Cohort (80)	14.0	1120	4	0.4	No	Patients > 10 yr at diagnosis	Bharucha et al., 2015 [27]
1972–2014	8.4	MC, nat Cohort (155)	10.9	1766	39 (Under FU = 27)	Total incl cSCD: 2.4 *FU-grp:* <1999:1.8 ≥1999-:1.1	**Yes**	None	Östman-Smith et al., 2017 [19]
1974–2016	12.2	SC, TC (100)	9.2	920	19 (LAE)	2.1	No	RAS-HCM	Maurizi et al., 2018 [20]
1970–2017	11	MC, TC (1024)	5.3	5984	89 (MACE)	1.5	No	RAS-HCM	Norrish et al., 2019 [28]
?–2017	9.8	MC, TC (572)	5.0	2855	53 (LAE)	1.9	No	RAS-HCM (PRIMaCY)	Miron et al., 2020 [18]
?–2017	13.8	MC, TC (285)	4.9	1400	22 (LAE)	1.6	No	RAS-HCM (ShaRe)	Miron et al., 2020 [18]
1972–2016	10.9	MC, Nat Cohort (151)	11.6	2008	ns-HCM = 27 RAS-HCM = 6(LAE)	ns-HCM = 1.8 RAS = 1.7	No	Patients presenting with arrest	Östman-Smith et al., 2021 [29]

Abbreviations: Era = era of patient recruitment; D = at diagnosis; yr = years; n = number of patients; SCD = sudden cardiac death; incl comm SCD = inclusion of patients presenting with SCD in community as first symptom; SC = single centre; MC = multi-centre; TC = tertiary centre; FH = family history; ns-HCM = non-syndrome-associated HCM; NST = no specific therapy; HDBB = high-dose beta-blocker therapy; RAS = HCM associated with Noonan syndrome or other RASopathy; Total incl cSCD = including patients presenting with SCD in community; FU-grp = patients diagnosed and under follow-up; LAE = lethal arrhythmic event (SCD, cardiac arrest, ICD appropriate discharge); MACE = malignant arrhythmia cardiac event (SCD, cardiac arrest, ICD appropriate discharge, sustained ventricular tachycardia); RC = regional geographic cohort; Nat cohort = national cohort; phenocopies = secondary HCM, due to for example storage disorder or Friedreich´s ataxia.? = Age at diagnosis not given in the article, only age at electro-physiological study.

In Table 1, studies from which “annual” mortality rates can be calculated are listed in date order, with the earliest at the top of the table. The studies report cases with primary HCM and do not include phenocopies (for example Danon, Pompe and Friedreich syndromes) unless specified. As shown, the “annual” mortality rate is overall lower in studies that only report SCD as an end-point in patients under follow-up (around 1.3%), as compared with studies that include deaths from previously undiagnosed cases in the community (2.4–3.7%). Later studies on patients under follow-up that include also resuscitated cardiac arrest and appropriate ICD-interventions have higher event rates, between 1.6–2.6%, although there is a suggestion of an era effect in at least one study with a rate of 1.1% in the more recent patients. Two small studies have conspicuously lower rates. A study from the Australian Cardiomyopathy Registry had a very low median age at diagnosis, only 0.45 yr, and recruited only patients below 10 years of age at diagnosis; they reported an SCD rate of only 0.4% [27]. The low rate is not surprising if one looks at Figure 1, and realizes that many of the patients in the Australian cohort have only just reached the ages of higher risk. A second study, by Decker et al. 2009 [24] also reported a low SCD rate of 0.5%, here the median age at diagnosis was 10.6 y. The unusual feature of this study was that virtually all patients received pharmacotherapy, and 86% of patients received beta-blocker therapy, a much higher proportion than in any other study. High-dose beta-blocker therapy was also associated with a low risk of SCD and improved survival in an earlier study [23]. Furthermore, several later retrospective studies have found beta-blocker dose to be associated with a lower risk of SCD even on multivariate Cox-hazard regression [19,29], and this might explain the low SCD-rates found in the study by Decker et al. 2009 [24]. 

As regards the risk of SCD in *HCM associated with a RASopathy*, the data are very sparse, with one early study reporting no SCD occurring [30], but subsequently, four cases were reported among 26 patients with HCM associated with Noonan syndrome with lentiginosis, some of them adult [31]. As seen in Table 1, the rate of SCD has been the same as, or slightly lower than, that of patients with non-syndrome-associated HCM in the few studies where they have been separated out. A study of a pediatric national cohort of RASopathy HCM reported that among 27 patients with RASopathy-associated HCM, who survived infancy without heart failure death, there were six SCD (none of them associated with lentiginosis phenotype) in 343 patient-years (average annual risk 1.7%) [29]. The median age for SCD was 14.4 y, about the same as in non-syndromal HCM [29]. Thus there is no current evidence that those patients with RASopathy-associated HCM that survive infancy without dying of heart failure are at significantly lower risk of SCD than non-syndromic HCM cases.

***Conclusion*** The risk of SCD in childhood-HCM is higher than in HCM diagnosed in adulthood, and the age range at highest risk is that corresponding to the pubertal growth spurt. The risk is highest in patients diagnosed already below 12 years of age, which is a powerful argument for carrying out cascade family screening of children of parents with HCM before the age of statistical risk increase, i.e., before 8 years of age.

## 3. Sex

As HCM is generally inherited as an autosomal dominant, one would expect an even sex representation in HCM cases in childhood; however, studies in familial HCM have suggested that the penetrance to expressed phenotypic disease is both age and gender-related with higher penetrance in males at least below 40 years of age [32,33]. Even though there is a substantial proportion of sporadic new mutations in studies of childhood-HCM, around 31–38% in Swedish and British geographical cohorts [12,19] and between 47% and 52% in international tertiary centre collaborations [18,28], there is still a male predominance in all studies of childhood-HCM. In clinically diagnosed cases this ranges from 58–62% in geographical cohorts to 60–69% in tertiary centre studies [12,18,19,24,26,28]. Even in childhood-HCM detected by school screening, there is a 55% male preponderance [34]. The sex distribution of cases of SCD occurring in childhood-HCM very closely reflects the sex distribution in the case group in all the abovementioned studies. Consistent with that, sex has not been found to be a risk factor for sudden death either on univariate [12,26] or multivariate Cox-hazard regression [18,19,28]. 

***Conclusion*** All available evidence shows that sex is not a risk factor for sudden cardiac death in childhood-HCM.

## 4. Left Ventricular Hypertrophy

**Figure 2:**
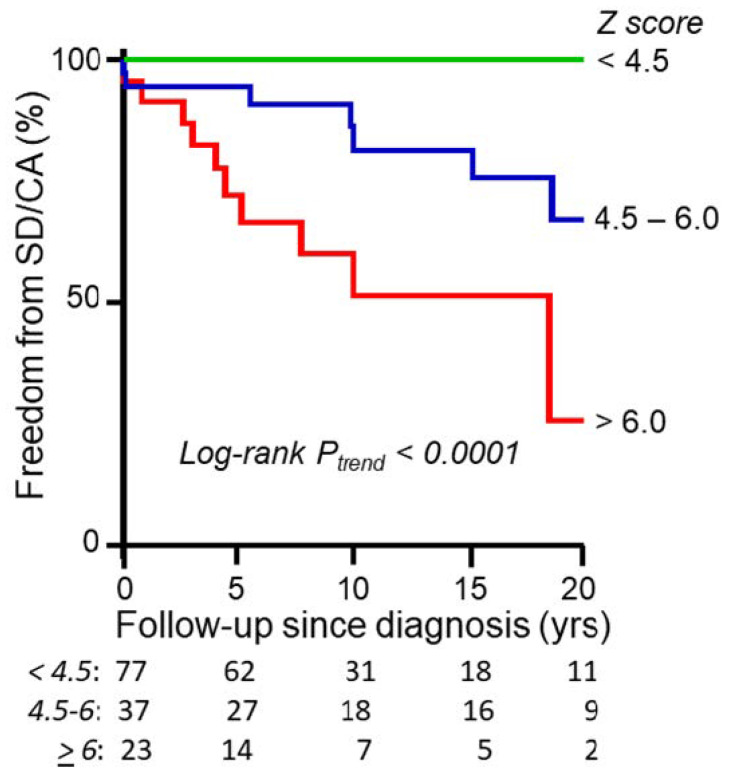
Illustrates Kaplan–Meier analysis of freedom from sudden death or resuscitated cardiac arrest in patients stratified according to the Detroit Z-score of maximum wall thickness at diagnosis, and illustrates a progressively increasing risk with increasing Z-score, with *p*-value for log-rank trend < 0.001. (Reprinted with permission from Östman-Smith et al. Open Heart 2017, 4, e000658. doi:10.1136/openhrt-2017-000658) [19], copyright article authors, 2017.

Maximal LV wall thickness was the first phenotypic characteristic to be found to correlate with the risk of SCD in adult HCM [35], and in the adult, the high-risk cut-off has been suggested to be ≥30 mm [36]. Maximal wall thickness cannot be determined on M-mode echocardiography alone and should be established by serial short-axis views using 2-D cardiac echocardiography. It is important to also measure apical wall thickness. In adult HCM-patients a maximal wall thickness ≥ 30 mm has been considered a high-risk cut-off, and it was suggested, but never verified, that a corresponding high-risk cut-off in childhood-HCM might be a wall thickness Z-score ≥ 6 [24]. Which Z-score should be used was never specified. Using ≥30 mm cut-off in childhood-HCM has a sensitivity of only 30% [19]. Only larger studies that have related maximal wall thickness either directly to body surface area [25], or indirectly as Z-score [18,19,28,29], or to normal value for age [9,19], or expressed it as a wall-to-cavity ratio, [9] have established a statistically significant correlation between increasing wall thickness and risk of an SCD or SCD-equivalent (resuscitated cardiac arrest, or appropriate ICD-discharge). There is a gradual increase in risk with increasing wall thickness [19,25], see Figure 2. Thus maximal wall thickness Z-scores have been incorporated as a continuous function in both recently suggested risk-calculation algorithms for childhood-HCM, HCM Risk-Kids (with a hazard ratio of 1.05 per unit increase in Z-score for five-year risk) [28], and PRIMaCY-SCD [18]. Of the incorporated risk factors in these algorithms, maximal wall thickness has the highest hazard ratio, and by far the strongest statistical association of any algorithm in HCM Risk-Kids [28]. Similarly, the interventricular septum Z-score appeared to have the steepest increase in the risk of parameters included in PRIMaCY-SCD [18]. In most, but not all childhood-HCM cases, the maximal wall thickness is localised in the interventricular septum. 

In the PRIMaCY study the degree of hypertrophy of the *posterior LV wall*, in most cases probably just a marker of generalized as opposed to localized cardiac hypertrophy, was incorporated in the risk-assessment algorithm in addition to septal thickness Z-score, as it improved the model. It did correlate with an increase in risk at least up to a Boston Z-score of 20. It was left undefined whether a Boston M-mode or 2-D Z-score was used for this analysis; the range of numbers suggests it was the 2D [18]. In already clinically high-risk patients implanted with an ICD, the LV posterior wall Z-score was significantly related to the risk of potentially lethal arrhythmia on multivariate analysis, with a hazard ratio of 1.02 per unit Z-score, and LV wall Z-scores ≥ 5 were defined as a high-risk cut-off; here, too, it was not specified which Z-score was used [37]. The definition of the Z-score matters hugely because different Z-scores give vastly different numerical values. As an example, the same maximal wall thickness measurement in mm gave a childhood-HCM cohort median value of 4.3 [IQR 2.6–5.8] using the Detroit M-mode Z-score [38], whereas the Boston body weight-only 2-D Z-score (as used in HCM Risk-Kids [28]) gave values of 9.6 [5.9–16.5], a difference of a factor of 2.2. The Z-scores of the patients suffering SCD-events differed by even more, 5.8 [4.1–6.9] for Detroit-score versus 16.7 [11.9–20.8] for Boston, different by a factor of 2.9 [29]! This suggests that these two Z-scores diverge more and more the higher the measurement value. Using the online Boston Z-score calculator https://zscore.chboston.org (accessed 14 March 2022) gives similar discrepancies between numerical values for Boston M-mode versus Boston 2-D Z-scores, with higher values for 2-D scores for the same mm measurement. Thus a simple change in the use of the type of Z-score is probably behind the apparent large shift in opinion as to what constitutes a high-risk degree of hypertrophy, from a Z-score ≥ 6 in American Heart Association (AHA) Guidelines 2011 and in ESC 2014 Guidelines [1,36], as compared to “around 20” in the 2021 AHA Guidelines [39]. Using the Detroit Z-score ≥ 6 is probably too high a cut-off, with a sensitivity of only 56% but high specificity, as compared to a lower cut-off of ≥4.5 having a higher relative risk of 9.9 and a sensitivity of 88% (over a mean total follow-up of 10.9 y) [19]. A cut-off of ≥4.5 also had a higher C-statistic than a cut-off of ≥6 for predicting SCD within 5 years, 0.79 versus 0.72 [29]. A Detroit Z-score ≥ 4.5 approximates to a Boston 2D-Z-score of ≥12.5 [29]. The use of the term “cut-off” should be understood as a practical tool, and must not obscure the fact that the degree of hypertrophy is not a binary function but a continuum of progressive risk, as also illustrated by the high event rate in patients with a Detroit Z-score ≥ 6, see Figure 2. The Z-score at diagnosis is often not the same as that during later follow-up as there is strong evidence for progression of left ventricular hypertrophy being common during later childhood, particularly during the pubertal growth spurt [40]. Thus the quantification of hypertrophy by using Z-scores needs to be continued all through childhood and adolescence at regular intervals, and with most follow-up appointments, in order to keep the risk profile up to date. 

***Conclusion*** Left ventricular hypertrophy is a very major risk factor with a steep increase in risk with an increase in the degree of hypertrophy, probably best quantified by the use of Z-scores, but great care must be taken to use the same Z-score as the risk-assessment instrument used for reference.

## 5. Left Ventricular Outflow Tract Obstruction

Among adult HCM-patients, left ventricular outflow obstruction (LVOTO) is a recognized risk factor for morbidity, total mortality and SCD [1,41]. In childhood-HCM, there are conflicting reports. Early studies with few SCD end-points found no evidence that LVOTO was a significant predictor [22,24], although Ziolkowska et al. found it to be a significant risk factor for combined arrhythmia + heart failure death end-points [26]. Notably, however, in a multi-national study with a mean follow-up of 7.0 y (and including patients presenting with sudden death) patients who had suffered SCD had a much higher prevalence of outflow tract obstruction (79%) compared with long-term survivors (46%), or the total combined group (55%) [12]. Similarly, among the 1024 patients followed in the HCM Risk-Kids study cohort, with a mean follow-up of 5.3 y, 31.7% of subjects with an SCD-event had an LVOT-gradient ≥ 30 mmHg, versus only 18.4% in the total study cohort [28]. A Swedish national cohort study with 32 end-points found that an LVOT-gradient > 20 mmHg at diagnosis was a significant risk factor for SCD on univariate Cox-hazard (*p* = 0.009), and it remained a risk factor in the multivariate analysis [12]. If at the last visit the LVOT-gradient was >20 mmHg the relative risk for SCD was 3.7, and if it was >50 mmHg it was 6.6 (*p* = 0.004, and *p* < 0.001, respectively). However, in the HCM Risk-Kids multi-national study cohort, in spite of the higher prevalence of LVOT-gradients at diagnosis in the patients with SCD-events, the analysis suggested possibly a marginal reduction in risk (hazard ratio 0.99 [0.99–1.00], *p* = 0.10, so not definitely significant). LVOT-gradient at diagnosis was, nevertheless, incorporated as a parameter that reduced risk in the HCM Risk-Kids algorithm [28]. In PRIMaCY-SCD, the analysis suggested a slightly more complicated relationship, with risk appearing to increase between gradients of 30 mmH and 80 mmHg, and then fall again when gradients rose above 80 mmHg [18]. There were a significant number of patients with missing data for this parameter in both studies, 15% in HCM Risk-Kids, and 29% in PRIMaCY. In both studies, imputed values were used to substitute for these missing values. How can one make sense of these conflicting results? It seems inherently unlikely that the presence of an outflow-gradient should be protective in early life, only to become a risk factor after 16 years of age. As we are here considering only outflow-gradient present at diagnosis, the apparent protection could well be due to a confounding effect of patients with outflow-gradients having more intensive therapy, with higher doses of beta-blockers, or beta-blockers combined with disopyramide (which also has beneficial effects on diastolic function), as compared to non-obstructive patients. This possible confounding role of beta-blocker therapy and beta-blocker dose is supported by increasing beta-blocker dose being associated with a significant reduction in risk of SCD in multivariate Cox-hazard analysis in a Swedish national cohort [19,29]. A protective effect of beta-blocker therapy on total cardiac mortality has now also been found in several large studies in adult HCM, in the USA, Taiwan and Sweden, confirming that beta-blocker therapy is a plausible confounder [42,43,44]. The same confounder could also be responsible for childhood ICD-recipients with LVOTO receiving fewer appropriate shocks [37]. There might also in addition be a question of duration of un-relieved LVOTO to explain the different effects in adult and pediatric HCM. Whether or not the patient has had effective surgical or interventional treatment controlling LVOTO, could be another confounder. One Swedish national study certainly found that both a larger beta-blocker dose and a myectomy procedure were associated with a reduction in the risk of SCD on multivariate analysis [29].

**Figure 3:**
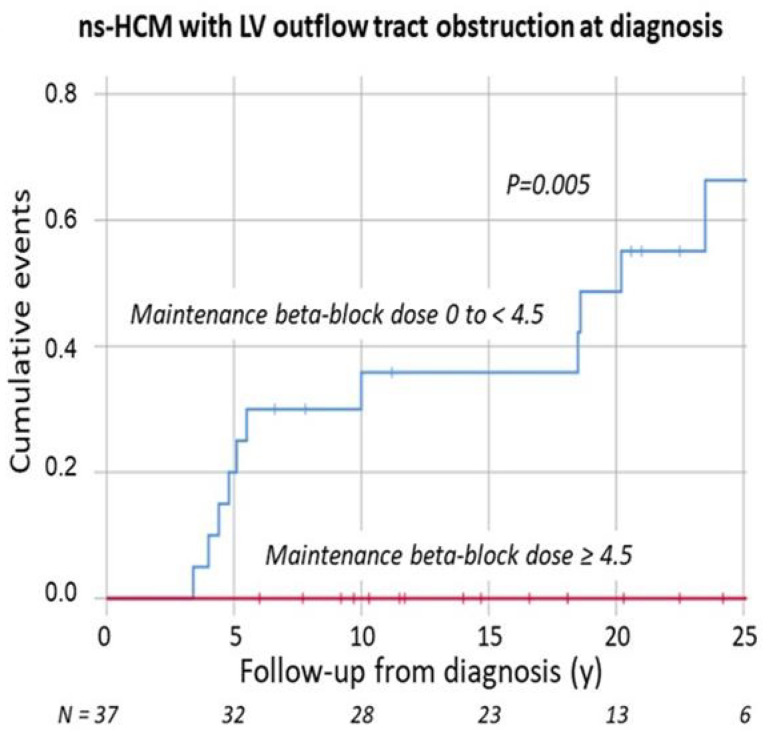
Kaplan–Meier analysis of accumulation of SCD-events in pediatric patients with non-syndrome-associated HCM and an outflow-gradient >20 mmHg at diagnosis, stratified depending on therapy after diagnosis, the curve in red illustrates patients with a propranolol-dose equivalent of ≥4.5 mg/kg/day (no events), and the blue curve patients with no beta-blocker therapy, or a dose equivalent to <4.5 mg/kg/day. Ticks on curve indicate patients being censored alive, and follow-up duration is measured in years. N indicates number of patients left in curve at different follow-up duration. (Reprinted with permission from Östman-Smith et al. Acta Paediatrica 2021, DOI: 10.1111/apa.16045 Supplementary Figure S1) [29], copyright authors, 2021.

The impact of freedom from SCD in patients with an LVOT-obstruction at diagnosis of treatment with a propranolol-equivalent dose of ≥4.5 mg/kg/day as compared to no therapy or low dose, is shown in Figure 3, illustrating the significantly superior freedom from events in patients with effective beta-blockade (*p* < 0.005).

Furthermore, the risk might be over a longer-term than five years, and what constitutes the risk is an LVOT-gradient that is not sufficiently controlled on later follow-up. This hypothesis is supported by residual LVOT-gradient on follow-up being a much stronger predictor of SCD on Cox-hazard regression than LVOT-gradient at diagnosis: the C-statistic of residual LVOT-gradient on follow-up was 0.65 (ns) for SCD within 5 years, 0.70 for SCD within 10 years, and 0.79 (*p* < 0.001) for SCD within a median follow-up of 13.4 years [29]. The size of LVOTO has been shown to correlate with the degree of delayed late enhancement on MRI in childhood-HCM, suggesting that it might accelerate myocardial fibrosis [45]. It is the five-year risk, and only the gradient at diagnosis, that has been assessed in HCMRisk-Kids and PRIMaCY-SCD [18,28].

***Conclusion*** The evidence supports the view that the *persistence* of a significant size LVOTO-gradient after appropriate therapy might be considered a risk factor to be considered in the decision about possible ICD-implantation. Further research with longer follow-up, using the gradient after therapy has been commenced and not the gradient at diagnosis, might help quantify risk more clearly. The use of imputed values should be avoided in risk factor research.

## 6. Diastolic Myocardial Function—Restrictive Physiology

Experimental studies in many models suggest that the primary abnormality in familial HCM is impaired myocardial function leading to compensatory LV hypertrophy [46]. In familial HCM, abnormalities in diastolic function detectible by tissue Doppler often precede a clearly abnormal degree of hypertrophy, and diastolic function predicts which phenotype-negative mutation carriers will develop HCM [47]. Different types of impairment of diastolic function have been described as causing “restrictive physiology”. As early patient cohorts lack tissue Doppler measurements, left atrial enlargement has often been used as a surrogate indicator of restrictive physiology. An early study used either LA:aortic ratio ≥ 1.5, trans-mitral flow E:A ratio ≥ 3, or tissue Doppler septal trans-mitral early-diastolic (E) to early-diastolic septal tissue Doppler (e) velocities as E:e ratio ≥ 10, to define “restrictive physiology”. They found that the presence of restrictive physiology conferred a 3.8-fold hazard of combined end-point of death or aborted SCD within a 10-year follow-up period [48]. A smaller study with 80 childhood-HCM subjects, and only 14 of a combined end-point (death, aborted SCD, and presence of ventricular tachycardia), found values in the latter of E:e ratio 13.7 [12.3–15.1; *p* < 0.001 versus patients without events], and septal e-velocity 7.3 ± 2.4 cm/s (*p* = 0.01) [49]. Diastolic dysfunction and maximal wall thickness (*p* = 0.002) were the only significant clinical characteristics, whereas left atrial volume indexed to body surface area did not reach significance [49]. Significantly reduced velocities of trans-mitral E-flow, 63 [46–91; *p* = 0.04] cm/s, were found in childhood-HCM-patients with a combined heart failure death and arrhythmic end-points, as compared to 85 (74–97) cm/s in patients without end-points, but the difference did not reach significance if only arrhythmic end-points were considered [26]. Levels of B-type natriuretic peptide (BNP) correlate with diastolic dysfunction in childhood-HCM [50]. In adult HCM-patients, BNP levels >312 pg/mL have been reported to be a risk factor for SCD [51], but BNP levels have not been studied in relation to SCD-risk in childhood-HCM. Studying atrial conduit function by means of CMR techniques using feature tracking technology is a new technique available to evaluate the diastolic function, and a recent study in childhood-HCM demonstrated a significantly reduced passive atrial emptying fraction in patients with an end-point of SCD or aborted SCD [52]. Systolic dysfunction, as measured by systolic deformation and strain techniques is present in children with HCM, but there are as yet no studies to define its role in risk assessment [53], or if it is an independent risk factor from measurements of the degree of hypertrophy.

***Conclusion*** Impaired diastolic function appears to be a potentially important and independent risk marker, but currently available studies are too small to quantify the relative increase in risk precisely.

## 7. Left Atrial Enlargement

As illustrated above, left atrial (LA) enlargement has been used as a surrogate marker for restrictive physiology, but it can be caused by other factors such as mitral valve incompetence associated with dynamic left ventricular outflow obstruction with the systolic anterior movement of the mitral valve, and perhaps also by cardiomyopathy-expression in the atrial myocardium. However, even if it is multifactorial in origin, by virtue of this information often being available even in historical cohorts, it has been incorporated in the two pediatric risk-assessment algorithms published so far, HCM Risk-Kids and PRIMaCY-SCD [18,28]. In HCM Risk-Kids it was included as a preselected parameter, and in the derivation cohort, quantified as an LA diameter Z-score, it had a hazard ratio of 1.19 per unit Z-score (*p* < 0.001), but 34% of LA values were missing and had been imputed [28]. In a later analysis of a smaller (*n* = 356) subsection of the cohort with more complete data (18% missing LA Z-scores), the hazard ratio was lower, 1.03, and not statistically significant (whereas in contrast maximal wall thickness Z-scores hazard ratio 1.07 remained highly significant, *p* = 0.002). In the PRIMaCY-SCD derivation cohort (*n* = 572) LA enlargement correlated with increased risk and was incorporated in the final algorithm in addition to the ventricular wall thickness Z-scores, although these authors used imputed values for 21% of patients with missing LA Z-scores [18]. The assessment of LA enlargement is hampered by the fact that the LA diameter Z-score is not as accurate a measurement of enlargement as LA volume indexed to BSA by echocardiography, or ideally by CMR, but those types of volume measurements are not available in the large retrospective cohorts with sufficient end-points for multivariate analysis.

***Conclusion*** Left atrial enlargement is a useful marker for increase in risk, but whether it is an independent risk factor in relation to diastolic dysfunction, has not been established.

## 8. Electrocardiographic Phenotype

*ECG features correlating with SCD.* The features of the ECG reflect the speed and homogeneity of the myocyte depolarisation and repolarisation process, where it is known that inhomogeneity will lower the threshold for ventricular arrhythmias to occur. From a Mayo clinic study on 2485 HCM-patients, it was reported that there was no SCD among the 135 patients with normal ECGs [54]. It might have been assumed that this simply reflected that those patients had particularly mild hypertrophy, but it has been shown repeatedly in childhood-HCM that ECG amplitudes and amplitude x duration products were independent risk factors for SCD from the degree of ultrasound-measured hypertrophy [9,19]. Interestingly, some ECG characteristics are inheritable rather than directly related to myocardial mass [55], and have been localized to four distinct gene loci [56]. Already in 1999, it had been noted that large Sokolow-Lyon index voltages were associated with higher mortality in pediatric HCM [23], but more widespread research on ECG features in relation to risk for SCD has been hampered by the lack of archived ECGs in many large retrospective HCM-databases. A subsequent British–Swedish study established that in childhood-HCM a voltage sum of ≥10 mV in the six limb-leads (LLQRSS) was a risk factor independent of echocardiographic hypertrophy [9]. ST-segment depression was reported to be associated with SCD in adult HCM [57], and there followed a systematic study in adult HCM-patients to define features in resting ECG significantly associated with SCD. This study delineated QRS-axis deviation, T-wave inversion, in particular precordial T-wave inversion, dominant S-wave in V_4_, QTc, LLQRSS, and 12-lead QRS-amplitude x duration product as being features and measurements significantly associated with SCD [58]. 

*An ECG risk score system* was proposed based on the above findings, with point scores of 1 point or 2 points for morphological ECG characteristics, depending on the strength of statistical association. For ECG measurements, there is a range between 0–3 points depending on the measured value, as shown in Table 2.

**Figure 4:**
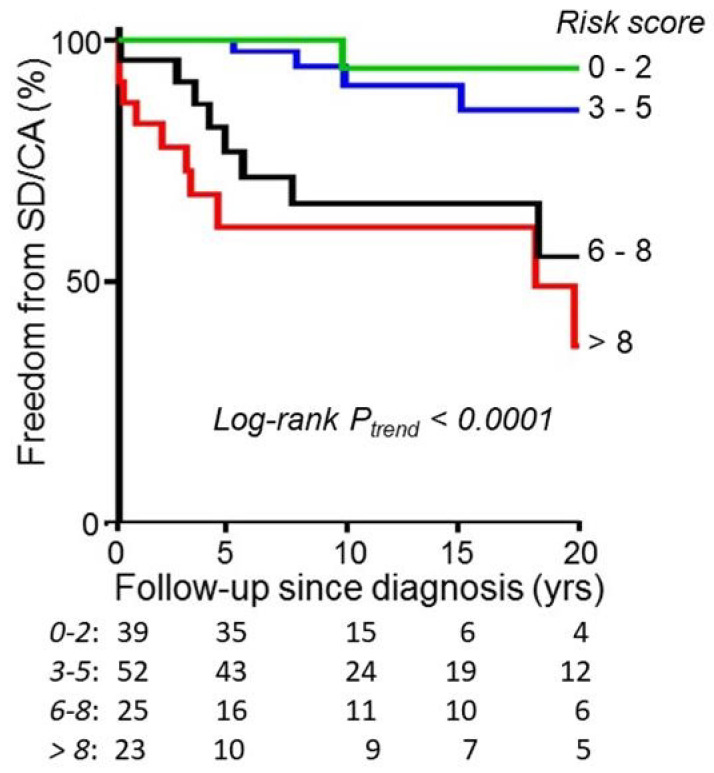
Illustrates Kaplan–Meier analysis of freedom from sudden death or resuscitated cardiac arrest in patients stratified according to the ECG risk score at diagnosis, and illustrates a progressively increasing risk with increasing risk score, with *p*-value for log-rank trend < 0.001. (Reprinted with permission from Östman-Smith et al. Open Heart 2017;4:e000658. doi:10.1136/openhrt-2017-000658) [19], copyright article authors, 2017.

When the ECG risk score was >5 points as calculated above (with the high-risk cut-off validated by boot-strapping), it gave a sensitivity for the cardiac arrest of 84%, and high specificity in adult HCM-patients < 40 years of age [58]. In a national Swedish pediatric HCM cohort, with a 10.9 y mean follow-up, which also included patients who presented with SCD or resuscitated cardiac arrest (who are known to be at particularly high risk), the same ECG risk score cut-off of >5 points conferred a very high relative risk of 46.5 [6.6–331] for SCD during later-follow-up, with a sensitivity of 97% [19]. The specificity for ECG risk score at the first visit was 69%, and the score at the last visit before the event was 80%, for the prediction of SD/CA in pediatric HCM-patients [19]. In a later study, excluding patients presenting with SCD or aborted SCD from the analysis and with a mean follow-up of 13.3 y, 40% of the population was test-positive [29]. Only including SCD in the first five years of follow-up as an end-point, an ECG risk score > 5 gave a specificity of 73% at diagnosis [29]. The risk for SCD is progressive with increasing value of ECG risk score as illustrated in Figure 4, and there is continued accumulation of SCD occurring even after five years of follow-up. 

Studies in the sub-set of the study population for HCMRisk-Kids with ECGs archived found a cut-off of >5 points only modestly predictive for a wider end-point of “freedom from major arrhythmia in the next five years” over a short follow-up of only 3.9 yrs. However, when the total ECG point-score was used, the study found a hazard ratio of 1.11 per point, higher than the hazard ratio for the LA Z-score of 1.03 in the same population [59]. A recent external validation of the ECG risk score cut-off >5 points in a tertiary centre childhood-HCM study group from Toronto, with a mean follow-up of 14.6 y, found a sensitivity of 95% and specificity of 56% for SCD in the first 5 years of follow-up [64]. All the above studies find very high negative predictive values for cut-off >5 points, between 97–100% [19,59,65,66]. QTC dispersion is an additional ECG feature that has been reported to have a significant correlation with SCD in childhood-HCM, with a hazard ratio of 1.6 per 20 ms increase [22], or a cut-off of 0.055 being associated with arrhythmic end-point [26]. There are, however, concerns about QTC dispersion measurements having much poorer reproducibility than QTc [67], and for that reason, it has not been included in the ECG risk score. A further strength of the ECG risk score is that it has been shown to be applicable to risk stratification in both familial HCM due to sarcomere protein mutations, as well as in HCM related to RASopathy syndromes [29,66].

***Conclusion*** The ECG risk score is not specific enough to be used as a risk indicator on its own, but because of easy availability, it is an excellent screening method for selecting cases that need accelerated further investigations, and to identify low-risk individuals already at the first out-patient visit. As it is a risk factor independent of the degree of hypertrophy, incorporating it in risk-assessment algorithms has the potential to improve their performance.

## 9. Myocyte Energy Deficit, and Its Importance for Ischaemia on Exercise

As shown above, ST-depression at rest, which is generally considered to commonly reflect ischaemia of at least sub-endocardial layers of the myocardium, is a risk factor for SCD. There are in addition particular features of the HCM-myocardium which constitute an enhanced risk for the myocyte to experience metabolic stress, which thereby also lowers the tolerance of the myocardium for the increased energy expenditure required during physical exercise.

*Cellular energy deficit.* An important finding in HCM secondary to the most common sarcomere protein mutations is that the myocardium shows evidence of a bio-energetic deficit with reduced cardiac phosphocreatine to adenosine triphosphate (PCr/ATP) ratios even in mutations carriers who have not yet developed cardiac hypertrophy [68]. The PCr/ATP ratio appears to be of clinical relevance as serial magnetic resonance studies have shown that PCr/ATP ratios < 1.44 are associated with more rapid progression of myocardial fibrosis as measured with late gadolinium enhancement (LGE) [69]. Obviously, if you have a poor PCr/ATP ratio even at rest, then during exercise there is a risk that mismatch of myocardial energy supply and cardiac work-load is enhanced, particularly as microvascular dysfunction is also common [70]. In the serial study, it was also found that impaired myocardial perfusion reserve on stress was associated with increased progression of LGE and that this in turn correlated with an increased number of events of a compound end-point including the new onset of non-sustained ventricular tachycardia [69]. In HCM, the coronary flow pattern in large vessels is pathological due to distal compressive deformation of intra-myocardial vessels resulting in a larger backward compression wave during systole, and also a smaller backward expansion wave, compared to controls. [71]. The presence of a severe left ventricular outflow tract obstruction causes additional deceleration of flow [71]. In young HCM-patients, microvascular dysfunction causing perfusion deficit during stress is often present in a larger area than the extent of myocardial fibrosis as delineated by late gadolinium enhancement (LGE) [70]. 

*ECG changes on exercise.* A more readily available method for looking for signs of myocardial ischaemia during exercise is the assessment of ECG changes provoked by exercise. Yetman et al. (1998) reported that childhood-HCM-patients with myocardial bridging on angiography had greater ST-depression on exercise testing and a significantly higher proportion of cardiac arrests or sudden death than patients without myocardial bridging [72]. In a subsequent study, the feature of ST-depression on exercise testing had a hazard ratio of 2.45 for SCD that did not quite reach statistical significance (*p* = 0.06) [22]. A more recent and larger study of a national cohort of pediatric HCM-patients showed that ST-depression ≥2 mm already at rest, not common in childhood-HCM, was present in 43.5% of patients with sudden cardiac death or cardiac arrest, and only in 8.4% of patients without arrhythmia events (*p* < 0.0001) [19]. ST-depression at rest may be more common in a tertiary centre population, where it was reported to occur in 16% of patients without arrhythmia events [59]. ST-depression during exercise testing conferred a relative risk of 5.7 [95%CI 1.9–17.4; *p* = 0.0035] of sudden death or cardiac arrest, with a positive predictive value of 56% and a specificity of 83% [19]. It is notable that the ST-depression on exercise testing in most of those patients was “silent”, and not reported to be associated with recognized chest pain. None of the other later pediatric studies has included ST changes during exercise testing in their analysis. ST-segment depression is probably more predictive in pediatric patients where it is rare than it would be in adult tertiary specialist centre populations, where it was present in 56% of a population with a mean age of 50 yrs [73]. However, even in adult HCM-patients ST-segment changes during exercise, in the form of ST-segment hump during exercise testing, were found to be a risk factor for sudden death [74]. For young children that cannot manage formal exercise testing a Holter recording during prescribed physical exercise or other excitement may also reveal ST-depression during high heart rates, but this has not been studied in relation to the risk of arrhythmia.

***Conclusion*** There are only a few, and small, pediatric studies of myocardial ischaemia during exercise as a risk factor so far, but all published findings point to ST-depression during exercise testing probably being a significant risk factor for SCD in childhood-HCM. The size of the increase in risk needs to be delineated in larger studies.

## 10. Myocardial Fibrosis and Scarring

Cardiac magnetic resonance imaging (CMR) has become a gold standard technique not only for the determination of myocardial mass but also for the assessment of the degree of myocardial scarring as delineated by late gadolinium enhancement (LGE). In early studies, the extent of LGE was quantified by the number of segments involved, and a small study reported the presence of LGE in 52% of subjects, and an apparent association of the number of segments involved with the presence of ventricular tachycardia [75]. A more detailed analysis can quantify LGE as a per cent of myocardial mass involved. In HCM-patients < 21 y of age, mean age of 14.6 y, 46% of patients had LGE, with a median extent of 3.3% of myocardial mass at the initial scan [76]. In those with repeat scans, there was significant progression to a prevalence of 52%, with a median extent of 4.3% of myocardial mass [76]. Evaluating a composite end-point including both severe symptoms and non-sudden cardiac deaths there was no difference in end-points between childhood-HCM-patients with LGE > 1% and ≤1% [77]. In a Chinese tertiary centre study, with children with severe HCM, LGE was detected in 73% with a mean extent of 10.4%. The absence of LGE was associated with significantly better freedom from a compound end-point of ventricular tachycardia and heart transplantation [78]. Thus, whereas extensive LGE appears to correlate with an increased amount of ventricular arrhythmia, there is still a lack of studies with an adequate number of SCD, or aborted SCD end-points, and therefore no good evidence about where a cut-off for high-risk might be best placed. There has been a suggestion that a cut-off >4%, when combined with HCM Risk-Kids improves the performance of the algorithm [79]. As shown by the studies above, however, >4% would include a large proportion of childhood patients. On the other hand, the AHA 2020 Guidelines suggest that “extensive” LGE is a risk factor for SCD, and tentatively suggest ≥ 15% as a possible cut-off, but stress the lack of consensus on this point [39]. Interestingly, the ECG risk score might be useful in deciding which patients need a CMR examination, as in a small study an ECG risk score < 3 points was associated with an absence of LGE in the majority of subjects, or at most a minor amount, <4% [65].

***Conclusion*** The evidence suggests that increased focal scarring identified by LGE increases the risk of ventricular arrhythmia, but as yet there are no pediatric studies that quantify the increase in risk of SCD or identify a reasonable cut-off.

## 11. Risk-Assessment Algorithms

Currently advocated risk-assessment algorithms include not only phenotypic factors but also some symptoms that are beyond the scope of this review; however, a brief comparison of the reported performance of the algorithms seems appropriate. It has been established that risk assessment based on only a binary assessment of adult HCM risk factors (ESC 2014 Guidelines, AHA2020 Guidelines) has less good performance than those which are based on the phenotypic risk markers being treated as continuous functions (e.g., HCM Risk-Kids, or PRIMaCY), see Table 3 for overview. 

The C-statistic value, from ROC-curve analysis, is commonly used for comparisons between predictive tests and is a measure of how well the test discriminates between the two outcomes, with 1.0 being a perfect prediction, and a value of 0.5 meaning no predictive power at all, thus a C-statistic needs to be significantly above 0.5 for the test to have any value at all. It is however a rather abstract concept, and what the clinician really wants to know is what proportion of test-positive individuals over a selected cut-off will actually suffer an SCD-event (positive predictive value) within five years, and how sure can I be that a test-negative individual will not have an event (negative predictive value)? Lastly, how many test-positive individuals will I have to consider an ICD for? Table 3 summarizes this information from studies evaluating different proposed risk-assessment instruments.

In the largest studied groups in Table 3 the observed prevalence of SCD-events in the five years after diagnosis range between 6.5–9.3% [18,28], and it is, therefore, a sign of insufficient specificity that advocated cut-offs end up with around 40–47% of patients test-positive. What is interesting in Table 3 is that the two single phenotypic marker assessments (maximal wall thickness Detroit Z-score, and ECG risk score) appear to perform at least as well as the multiple variable algorithms such as HCM Risk-Kids and PRIMaCY. However, the best performance comes by adding the HCM Risk Kids score to the ECG risk score, and only using values from age 7 y of age in cases diagnosed already in infancy. This underscores the fairly obvious point that risk status is not static from diagnosis, but needs to be continually reviewed during follow-up. 

***Conclusion*** Although newer risk-assessment algorithms for childhood-HCM outperform the old binary methods of risk assessment, they still have too low specificity to be able to be usable as the only decision instrument for implantation of an ICD. Attempts at combining ECG-phenotype markers with morphological markers have the potential to improve not only specificity but in particular sensitivity and negative predictive value.

## 12. Is Phenotype or Genotype More Important in Determining Risk?

In childhood-HCM, as in adulthood-HCM not associated with syndromes, the most common sarcomere mutations causing clinically overt HCM in an Italian study were *MYH7*, and *MYBPC3* [20]; however, the only group of mutations found to predict lethal arrhythmic events was the presence of a Troponin I or Troponin T-mutation (present in 13%). This was significant on univariate analysis, but *p* = 0.06 on multivariate analysis with the symptomatic state as the other predictor [20]. In a UK cohort, however, Troponin I or T-mutations only comprised 3% of mutation-positive childhood-HCM cases [10]. It has been reported that SCD can occur in patients with Troponin T-mutations and only minimal hypertrophy on echocardiography, but such individuals had very marked ECG changes that would have given very high ECG risk scores [82,83]. In their efforts to create a clinical risk prediction algorithm, the PRIMaCY-researchers tested two ways of including genetic information as predictors, either by increasing the risk for any type of positive genetic mutation diagnosis or by only giving risk-weighting to the presence of identified pathological *MYBPC3/MYH7*-mutations [18]. As compared with the phenotype-only algorithm, which as a continuous function had a C-statistic of 0.75, the ‘any positive mutation-approach’ had a C-statistic of 0.76 (clearly within the confidence limits of 0.75) [18]. The approach of risk-weighting only *MYBPC3/MYH7*-mutations had a C-statistic of 0.73 and was rejected as a model [18]. Widening the comparison to comparing risk in thick-filament associated mutations (e.g., *MYH7*, *MBPC3*), versus thin-filament mutations (e.g., Troponin T, alpha-tropomyosin or cardiac actin mutations), does not help in discriminating in risk for SCD either [84]. Research is hampered by only the more recent patients in large historic pediatric HCM cohorts used for risk factor studies having been genotyped. For example, in the PRIMaCY derivation cohort, only 54% of patients were genotyped, with less than one-third mutation-positive [18]. Similarly in the UK cohort, a minority, 107/433, of non-syndromal HCM-patients had identified mutations [10]. Thus, statistical power is poor. Furthermore, mutations in the same gene may have very different effects on the protein depending on if it is a mis-sense mutation, causing only a mild conformation change, or whether it is a truncating mutation resulting in a non-functioning protein; such factors have not yet been evaluated in risk assessment. Together with the large number of different genes implicated as causative in HCM, this creates such complexity as to make statistical risk analysis based on genetic findings very difficult. It is thus currently the situation that knowledge about the presence of a pathologic mutation does not predict the risk of lethal events, but only confirms that the patient may develop disease, even if it is not yet expressed. 

In adult HCM a *family history of SCD* is considered a risk factor for affected relatives, however, even very large studies in childhood-HCM have not found family history to be a significant risk factor for SCD in childhood-HCM [18,28], even when the analysis is restricted only to familial disease [19]. In the latter study, childhood-HCM-patients with a family history of SCD that had themselves died suddenly, were compared side-by-side with those individuals also with a family history of SCD, that had not themselves suffered an SCD-event, “survivors”. It was clear that the individuals suffering from SCD had a significantly different phenotypic expression. Among the eight suffering an SCD, most exhibited a severe phenotype with high-risk levels of hypertrophy and ECG changes already at diagnosis (maximal wall thickness Z-score *p* = 0.002, ECG risk score *p* = 0.006, respectively versus survivors), as compared to survivors without SCD where only 1/27 showed high-risk phenotypic features [19]. All patients that experienced SCD exhibited high-risk status before SCD occurred [19]. Whereas the presence of a particular mutation may confer a higher risk of developing a malign phenotype than a milder mutation, these findings suggest that it is the actual phenotypic expression caused by the mutation that carries the risk, not the mutation carriage as such.

As explained in the section “At what age is the risk highest?” patients with *syndrome-associated HCM* do actually also suffer SCD; however, published information on risk factors is very limited and from very small cohorts. They pinpoint a high limb-lead ECG voltage [9], a max wall thickness Detroit Z-score [66], and a combination of ECG risk score and HCM Risk-Kids ≥ 14 having a C-statistic of 0.91 [95%CI 0.79–1.00; *p* = 0.022] after seven years of age [29], as being significant risk factors. Table 4 below shows a meta-analysis of clinical findings reported from various studies and case reports.

Only some of the cases in Table 4, all with clinical Noonan-like dysmorphology, have had genetic testing performed. Among those tested, the mutations found include five cases with *PTNP11*-mutations associated with Noonan syndrome with lentigenosis, one case of *RAF 1*-mutation, one case of a *ALPK3*-mutation homozygote, and one with compound heterozygosity for *ALPK3*-mutations, both the latter ones had multiple dysmorphic features typical of Noonan syndrome. From this small sample, it appears confirmed that severe cardiac hypertrophy, a high ECG risk score, and prolonged QTc are common among syndrome-associated HCM-patients who suffer SCD. Larger studies are needed to determine if there are any differences as compared with risk factors in non-syndrome HCM-patients; however, it is notable that the majority of these cases had severe hypertrophy not only of the septum but also of the posterior LV wall, so generalized hypertrophy was common. Thus, even in syndrome-associated HCM, the phenotype appears the dominant influence on risk.

***Conclusion*** Both in non-syndromal childhood-HCM related to sarcomere protein mutations and in syndrome-associated childhood-HCM related to protein-kinase mutations, it is the phenotypic expressions of disease that gives the clearest indication of the risk of SCD.

## 13. Discussion

### 13.1. Current Knowledge Gaps 

One reason for the sub-optimal performance of the composite algorithms may be because they include parameters where there is a risk of substantial measurement errors. For left atrial diameter the measurement in the same patient can vary considerably depending on the angle of measurement and body position. For measurements of the left ventricular outflow tract gradient the actual velocity can vary considerably even within one day depending on the physiological state of the patient, it can also be difficult to align perfectly with the angle of outflow velocities. Furthermore, as soon as the velocity is too high for pulsed Doppler, so that continuous wave Doppler has to be used, there is always the danger that velocities of the mitral incompetence jet may get picked up and give a falsely high reading. This misinterpretation can be avoided by actually also measuring the velocity of the mitral incompetence jet to assess left ventricular pressure, but whether that has been conducted or not is hardly ever documented in old patient records. A critical analysis should be instituted, as to whether these two parameters actually add any meaningful discrimination in the current algorithms, even though they remain related to risk. 

In addition to ECG phenotypic scoring, there are two further highly interesting potential risk markers that might be included instead, for which more studies are needed, to nominate potential cut-offs and to evaluate the hazard ratios with those cut-offs. Those are diastolic dysfunction, most reproducibly measured by tissue Doppler and septal e-wave velocities and E:e-velocity ratios, and degree of myocardial scarring, ideally quantified as per cent myocardium with LGE on CMR. 

The large studies that aimed at constructing risk algorithms excluded all patients that had presented with a resuscitated cardiac arrest, presumably because the aim was to measure an “average” risk of a subsequent SCD-event. This approach makes those studies comparable to each other; however, it does lose informative patients with end-points that could help to define hazard ratios of individual risk factors, and perhaps future studies should include those high-risk patients in order to clarify the ranking between putative risk factors.

A significant gap in current knowledge is risk stratification for patients with HCM associated with a RASopathy-like syndrome, or RASopathy-like dysmorphology. They are specifically excluded from current international guidelines, as well as the application of the HCM Risk-Kids and PRIMaCY risk-assessment algorithms. This has resulted in very few pediatric cardiologists referring children with RASopathy-associated HCM for a primary prophylactic ICD, with the majority of existing ICD-systems in RASopathy patients being implanted only after resuscitated cardiac arrest [88]. Yet multiple reports documents that they do suffer SCD [9,22,85,86,87], and among those that survive infancy without dying from heart failure, the SCD rate was 15% of those survivors between their 8th and 18th birthday, thus a rate similar to non-syndromal HCM [29]. There were only six SCDs in RASopathy HCM in the latter study, so statistical power was limited but, as with non-syndromal HCM, adding HCM Risk Kids score to ECG risk score, and only using values from age 7 y of age showed the best discrimination, with a C-statistic of 0.91 [0.79–1.00] and good discrimination also of longer-term risk [29]. International collaborations are needed to collect sufficiently large populations for a more detailed analysis of whether RASopathy patients need a separate risk algorithm or not.

### 13.2. Future Directions

A weakness in both HCM Risk-Kids and PRIMaCY, and their external validation studies, is that the duration of follow-up is short, 3.5–5.3 y, meaning that at most half, but often considerably less, of the survivors have actually reached the time cut-off of 5 yrs. This means that patients censored alive before 5 years of follow-up may skew results [18,28]. Only the two studies that have fewer patients have long enough follow-up so that there are no survivors with a follow-up <5 y [29,64], and consequently have a total of patient-years that equal or exceed some of these ostensible larger studies. For future studies, one could wish that authors only included survivors with at least 5 y of follow-up and that they did not impute values for missing data, as that risks simply reinforcing old perceptions about risk parameters. Furthermore, potential confounders such as beta-blocker dose, and other interventions to reduce outflow tract obstruction should be included in multivariate analysis to define risk factors. To quantify risk over a 10-year follow-up should also be attempted, as performed recently [29], as the life span of an ICD-generator exceeds five years. 

**Figure 5:**
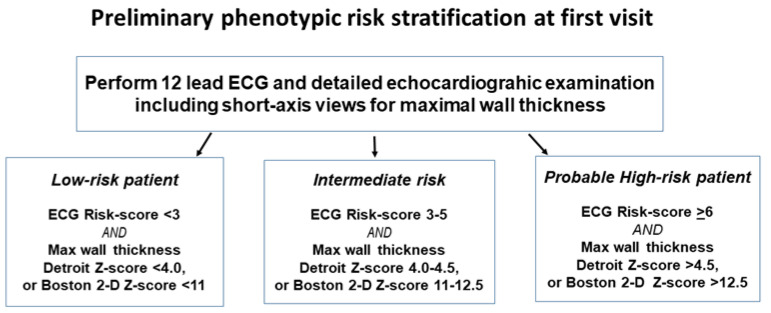
Illustrates how the investigative techniques available at most out-patient assessments could be used for an initial risk stratification that could guide further investigations.

Recent studies have started to include ventricular tachycardia as part of a compound end-point, but neither those, nor appropriate ICD-discharges, are really equivalent to an SCD, so might mislead [89]. Instead, it would be more productive to include the clinical data from patients presenting with resuscitated cardiac arrest as the first disease manifestation, in order to increase end-points with really hard data. This should help identify the best risk factors by hazard ratio in order to attempt to construct new risk algorithms with higher specificity, perhaps only based on the phenotypic characteristics, and then treat non-sustained ventricular tachycardia on Holter and syncope as red flag events rather than as part of the algorithm. To include syncope in the algorithm, with a hazard ratio as high as 7.23 as in PRIMaCY (based on tertiary centre patients) certainly created a large amount of the false positives in our external validation. It seems a very high hazard ratio as compared with hazard ratios from other reported pediatric childhood-HCM studies of 0.7 (in a geographical cohort) [19], 2.1 [20], and 1.48 [28] respectively, as well as a meta-analysis in adult HCM which found a significant association in only four out of 10 studies, with three of those four positive ones having hazard ratios 1.5–2.5 only [90]. A proposed scheme for gradual patient assessment, based on initial categorization using only ECG- and LV maximal wall thickness findings to guide the urgency of further examinations, has been proposed but not formally evaluated in other patient cohorts [29]. The initial step in the proposed preliminary risk assessment, which could be used to assess the urgency of further investigations to more definitely assess risk status, is shown in Figure 5.

More detailed risk assessment, and the calculation of HCM Risk-Kids and PRIMaCY-scores, require results from 24 h Holter monitoring, and the initial categorization in Figure 5 can serve as a guide as to how urgently 24 h Holter ECG, and potentially CMR with LGE, and exercise testing, needs to be carried out. A proposed flow-chart that recommends how to proceed from this particular initial categorization is available in an open access publication [29]. When the ECG risk score and maximal wall thickness category of a patient are in two different risk categories, it would be appropriate to initially follow the pathway of the highest risk category.

## 14. Conclusions

Risk-stratification algorithms for sudden cardiac death remain ‘a work in progress’ as they overestimate risk substantially without having a high sensitivity. To include additional phenotypic parameters, which can be reproducibly measured, might improve algorithm performance. Such measures include ECG-markers, e.g., ECG risk score (remembering the very high negative predictive value), tissue Doppler diastolic function measurements, and quantification of myocardial scarring on CMR. Until that work has been achieved, the latter three parameters provide clinical findings that can be of use, in conjunction with per cent risk calculated from current pediatric risk-assessment algorithms, in the difficult clinical decision as to which childhood-HCM-patient would benefit from an ICD-implantation.

## Figures and Tables

**Table 2:** Calculation of the ECG risk score.

Morphological Characteristics			Ped HCM Refs	Adult HCM Refs
		Points		
Deviation of QRS-axis present	Yes	1	[19]	[58]
Pathological T-wave-inversion ≥1 mm			[19]	[58]
present *In limb-lead*	Yes	1	[19]	[58]
*In precordial lead*	Yes	2		
*In both limb-lead and precordial lead*	Yes	2	[19,59]	[58]
ST-segment depression ≥ 2 mm present	Yes	2	[19]	[57,58]
S-wave greater than R-wave in lead V4	Yes	2	[19]	[58]
**ECG measurements**				
Six limb-lead QRS-amplitude sum in mV	0–7.6 mV ≥7.7–9.9 mV ≥10.0–11.9 mV ≥12.0 mV	0 1 2 3	[9,12,19]	[58]
12-lead QRS-amplitude x duration product mV.sec	0–2.19 mV.sec ≥2.2–2.49 mV.sec ≥2.5–2.99 mV.sec ≥3.0 mV.sec	0 1 2 3	[19]	[58]
QTc (Bazetts formula)	<440 msec	0		
	≥440 msec	1	[19,25,60]	[58,61,62]
Maximal total score		14 points		

Abbreviations: Ped HCM Refs = references showing association with risk for SCD in pediatric HCM-patients; Adult HCM refs = references showing association with risk for SCD in adult HCM-patients; Measuring principles: for limb-lead sum see Östman-Smith et al., 1999 [23], for 12-lead amplitude x duration product see Molloy et al., 1992 [63].

**Table 3:** Comparison of ability of parameters recorded at diagnosis to predict SCD or SCD-event occurring within five years, C-statistic with [95% CI].

Parameter	C-Statistic [95%CI]	Sens	Spec	PPV	NPV	%TP	Mean FU	Age at D	End -Pts	Patient Years
**Side-by-side comparison in the same cohort [29]**										
ESC ≥ 2RF	0.66 [0.47–0.85]	45	86	28	93	17	13.4	10.9	11	1474 [29]
AHA ≥ 1RF	0.55 [0.37–0.73]	55	56	13	91	45				
Max wth Det.Z-sc ≥ 4.5	0.79 [0.66–0.92]	90	68	24	98	38				
ECG Risc ≥ 6	0.87 [0.80–0.94]	100	73	31	100	40				
HCMRiskKids ≥ 6 (ext validation 1)	0.69 [0.64–0.89]	73	65	22	95	39				
HRK+ECGri ≥ 14	0.82 [0.68–0.96]	82	82	38	97	26				
7plHRK+ECGri ≥ 14	0.90 [0.83–0.96]	100	77	38	100	32				
**Studies with evaluation of single parameter cut-offs**										
HCMRiskKids ≥ 6% (compl values cohort)	0.69 [0.66–0.72]	76	58	12	97	44	5.3	11	34	3005 [28]
HCMRiskKids ≥ 6% (ext.validation 2)	0.70 [0.60–0.81]	74	73	14	98	30	3.5	12.3	23	1474 [80]
PRIMaCY ≥ 9% (ext validation 2)	0.66 [0.49–0.84]	82	52	20	95	40	10.6	10.6	11	1463 [81]
ECG Risc ≥ 6 (external validation)	0.76 [?]	95	56	28	99	47	14.6	?	22	2102 [64]
**Studies evaluation parameters as continuous functions**										
Max wthDet Z-score	0.79 [0.65–0.92]						13.4	10.9	11	1474 [29]
ECG risk score	0.91 [0.85–0.97]						13.4	10.9	11	1474 [29]
HCMRisk-Kids (ext validation 1)	0.77 [0.62–0.93]						13.4	10.9	11	1474 [29]
HCMRisk-Kids (ext validation 2)	0.75 [0.52–0.97]						3.5	12.3	23	1474 [80]
PRIMaCY (derivation cohort)	0.75 [?]						5.0	9.8	53	2855 [18]
PRIMaCY (ext validation cohort1)	0.71 [?]						4.9	13.8	22	1400 [18]
PRIMaCY (external validation 2)	0.71 [0.51–0.90]						10.6	10.6	11	1463 [81]

Abbreviations: C-statistic = area under the ROC-curve; Sens = sensitivity; Spec = specificity; PPV = positive predictive vale; NPV = negative predictive value; %TP = percent of patients that are test-positive above cut-off; FU = follow.up (y); Age at D = age at diagnosis (y); End-pts = end-points; ESC = ESC2014 Guidelines; RF = risk factors; AHA = AHA2020 Guidelines; Max wth Det.Z-sc = maximal wall thickness Detroit Z-score; ECG Risc = ECG risk score; HRK+ECGri = Sum of HCM Risk-Kids score plus ECG risk score; 7plHRK+ECGri = Sum of HCM Risk-Kids score plus ECG risk score at age 7 y if infant diagnosis, or at D if later.? = information not included in the article.

**Table 4:** Meta-analysis of clinical findings in reported syndrome-associated HCM with sudden cardiac death.

Parameter	Age at Diagnosis (yrs)	Age at SCD (yrs)	Detroit Z-Score (M-mode)	Boston Z-Score (2-D)	Max Wth (mm)	Post LV Wall Detroit Z-Score n = 7	QTc (ms) n = 12	Limb-Lead QRS-Sum (mV) n = 8	ECG Risk Score n = 8
Median	7.2	13.2	6.8	27.4	28	6.1	478	17.0	11
IQR	0.2–11.5	9.9–26	6.1–7.8	20.8–36	24–33	4.9–6.8	439–500	11.4–37	8–11
Range	0.01–17	2.0–42	4.7–8.7	13.3–43	17–40	2.7–8.9	408–528	10.9–42	7–13

*n* = 13, data from references [9,19,31,85,86,87]. Abbreviations: Max Wth = maximal wall thickness; PostLV = posterior left ventricular; Limb-lead QRSsum = sum of R-wave plus S- (or if deeper Q)-wave amplitudes of all six limb-leads.

## Data Availability

Not applicable.

## References

[1] Elliott P.M., Anastasakis A., Borger M.A., Borggrefe M., Cecchi F., Charron P., Hagege A.A., Lafont A., Limongelli G., Mahrholdt H. (2014). 2014 ESC Guidelines on diagnosis and management of hypertrophic cardiomyopathy: The Task Force for the Diagnosis and Management of Hypertrophic Cardiomyopathy of the European Society of Cardiology (ESC). Eur. Heart J..

[2] Reneman R., Schmitz J., Snoeckx L., Lambregts J., Lancée C. (1979). Differences in the echocardiographic dimensions of the heart between females and males. Echocardiology.

[3] Grandi A.M., Venco A., Barzizza F., Scalise F., Pantaleo P., Finardi G. (1992). Influence of age and sex on left ventricular anatomy and function in normals. Cardiology.

[4] Hada Y., Sakamoto T., Amano K., Yamaguchi T., Takenaka K., Takahashi H., Takikawa R., Hasegawa I., Takahashi T., Suzuki J. (1987). Prevalence of hypertrophic cardiomyopathy in a population of adult Japanese workers as detected by echocardiographic screening. Am. J. Cardiol..

[5] Maron B.J., Gardin J.M., Flack J.M., Gidding S.S., Kurosaki T.T., Bild D.E. (1995). Prevalence of hypertrophic cardiomyopathy in a general population of young adults: Echocardiographic analysis of 4111 subjects in the CARDIA study. Circulation.

[6] Arola A., Jokinen E., Ruuskanen O., Saraste M., Pesonen E., Kuusela A.L., Tikanoja T., Paavilainen T., Simell O. (1997). Epidemiology of idiopathic cardiomyopathies in children and adolescents. A nationwide study in Finland. Am. J. Epidemiol..

[7] Nugent A., Daubeney P., Chondros P., Carlin J., Cheung M., Wilkinson L., Davis A., Kahler S., Chow C., Wilkinson J. (2003). The epidemiology of childhood cardiomyopathy in Australia. N. Engl. J. Med..

[8] McKenna W., Deanfield J., Faruqui A., England D., Oakley C., Goodwin J. (1981). Prognosis in hypertrophic cardiomyopathy: Role of age and clinical, electrocardiographic and hemodynamic features. Am. J. Cardiol..

[9] Östman-Smith I., Wettrell G., Keeton B., Riesenfeld T., Holmgren D., Ergander U. (2005). Echocardiographic and electrocardiographic identification of those children with hypertrophic cardiomyopathy who should be considered at high-risk of dying suddenly. Cardiol. Young..

[10] Norrish G., Field E., McLeod K., Ilina M., Stuart G., Bhole V., Uzun O., Brown E., Daubeney P.E.F., Lota A. (2019). Clinical presentation and survival of childhood hypertrophic cardiomyopathy: A retrospective study in United Kingdom. Eur. Heart J..

[11] Marston N.A., Han L., Olivotto I., Day S.M., Ashley E.A., Michels M., Pereira A.C., Ingles J., Semsarian C., Jacoby D. (2021). Clinical characteristics and outcomes in childhood-onset hypertrophic cardiomyopathy. Eur. Heart J..

[12] Östman-Smith I., Wettrell G., Keeton B., Holmgren D., Ergander U., Gould S., Bowker C., Verdicchio M. (2008). Age- and gender-specific mortality rates in childhood hypertrophic cardiomyopathy. Eur. Heart J..

[13] Sugishita Y., Matsuda M., Iida K., Koshinaga J., Ueno M. (1983). Sudden cardiac death at exertion. Jpn. Circ. J..

[14] Maron B.J., Carney K.P., Lever H.M., Lewis J.F., Barac I., Casey S.A., Sherrid M.V. (2003). Relationship of race to sudden cardiac death in competitive athletes with hypertrophic cardiomyopathy. J. Am. Coll. Cardiol..

[15] Östman-Smith I., Rossano J.W., Shaddy R.E. (2013). Hypertrophic cardiomyopathy: Do sudden death prevention strategies in children differ between Europe and North America?. Curr. Opin. Cardiol..

[16] Maron B.J., Roberts W.C., Epstein S. (1982). Sudden death in hypertrophic cardiomyopathy: A profile of 78 patients. Circulation.

[17] Colan S.D., Lipshultz S.E., Lowe A.M., Sleeper L.A., Messere J., Cox G.F., Lurie P.R., Orav E.J., Towbin J.A. (2007). Epidemiology and cause-specific outcome of hypertrophic cardiomyopathy in children: Findings from the Pediatric Cardiomyopathy Registry. Circulation.

[18] Miron A., Lafreniere-Roula M., Steve Fan C.P., Armstrong K.R., Dragulescu A., Papaz T., Manlhiot C., Kaufman B., Butts R.J., Gardin L. (2020). A Validated Model for Sudden Cardiac Death Risk Prediction in Pediatric Hypertrophic Cardiomyopathy. Circulation.

[19] Östman-Smith I., Sjöberg G., Rydberg A., Larsson P., Fernlund E. (2017). Predictors of risk for sudden death in childhood hypertrophic cardiomyopathy: The importance of the ECG risk score. Open Heart.

[20] Maurizi N., Passantino S., Spaziani G., Girolami F., Arretini A., Targetti M., Pollini I., Tomberli A., Pradella S., Calabri G.B. (2018). Long-term Outcomes of Pediatric-Onset Hypertrophic Cardiomyopathy and Age-Specific Risk Factors for Lethal Arrhythmic Events. JAMA Cardiol..

[21] McKenna W.J., Deanfield J.E. (1984). Hypertrophic cardiomyopathy: An important cause of sudden death. Arch. Dis. Child..

[22] Yetman A.T., Hamilton R.M., Benson L.N., McCrindle B.W. (1998). Long-term outcome and prognostic determinants in children with hypertrophic cardiomyopathy. J. Am. Coll. Cardiol..

[23] Östman-Smith I., Wettrell G., Riesenfeld T. (1999). A cohort study of childhood hypertrophic cardiomyopathy: Improved survival following high-dose beta-adrenoceptor antagonist treatment. J. Am. Coll. Cardiol..

[24] Decker J.A., Rossano J.W., Smith E.O., Cannon B., Clunie S.K., Gates C., Jefferies J.L., Kim J.J., Price J.F., Dreyer W.J. (2009). Risk factors and mode of death in isolated hypertrophic cardiomyopathy in children. J. Am. Coll. Cardiol..

[25] Moak J.P., Leifer E.S., Tripodi D., Mohiddin S.A., Fananapazir L. (2011). Long-term follow-up of children and adolescents diagnosed with hypertrophic cardiomyopathy: Risk factors for adverse arrhythmic events. Pediatr. Cardiol..

[26] Ziolkowska L., Turska-Kmiec A., Petryka J., Kawalec W. (2016). Predictors of Long-Term Outcome in Children with Hypertrophic Cardiomyopathy. Pediatr. Cardiol..

[27] Bharucha T., Lee K.J., Daubeney P.E., Nugent A.W., Turner C., Sholler G.F., Robertson T., Justo R., Ramsay J., Carlin J.B. (2015). Sudden death in childhood cardiomyopathy: Results from a long-term national population-based study. J. Am. Coll. Cardiol..

[28] Norrish G., Ding T., Field E., Ziolkowska L., Olivotto I., Limongelli G., Anastasakis A., Weintraub R., Biagini E., Ragni L. (2019). Development of a Novel Risk Prediction Model for Sudden Cardiac Death in Childhood Hypertrophic Cardiomyopathy (HCM Risk-Kids). JAMA Cardiol..

[29] Östman-Smith I., Sjöberg G., Alenius Dahlqvist J., Larsson P., Fernlund E. (2021). Sudden cardiac death in childhood hypertrophic cardiomyopathy is best predicted by a combination of electrocardiogram risk-score and HCMRisk-Kids score. Acta Paediatr..

[30] Shaw A.C., Kalidas K., Crosby A.H., Jeffery S., Patton M.A. (2007). The natural history of Noonan syndrome: A long-term follow-up study. Arch. Dis. Child..

[31] Limongelli G., Pacileo G., Marino B., Digilio M.C., Sarkozy A., Elliott P., Versacci P., Calabro P., De Zorzi A., Di Salvo G. (2007). Prevalence and clinical significance of cardiovascular abnormalities in patients with the LEOPARD syndrome. Am. J. Cardiol..

[32] Charron P., Carrier L., Dubourg O., Tesson F., Desnos M., Richard P., Bonne G., Guicheney P., Hainque B., Bouhour J.B. (1997). Penetrance of familial hypertrophic cardiomyopathy. Genet. Couns..

[33] Terauchi Y., Kubo T., Baba Y., Hirota T., Tanioka K., Yamasaki N., Furuno T., Kitaoka H. (2015). Gender differences in the clinical features of hypertrophic cardiomyopathy caused by cardiac myosin-binding protein C gene mutations. J. Cardiol..

[34] Morimoto Y., Miyazaki A., Tsuda E., Hayama Y., Negishi J., Ohuchi H. (2020). Electrocardiographic changes and long-term prognosis of children diagnosed with hypertrophic cardiomyopathy by the school screening program for heart disease in Japan. J. Cardiol..

[35] Spirito P., Bellone P., Harris K.M., Bernabo P., Bruzzi P., Maron B.J. (2000). Magnitude of left ventricular hypertrophy and risk of sudden death in hypertrophic cardiomyopathy. N. Engl. J. Med..

[36] Gersh B.J., Maron B.J., Bonow R.O., Dearani J.A., Fifer M.A., Link M.S., Naidu S.S., Nishimura R.A., Ommen S.R., Rakowski H. (2011). 2011 ACCF/AHA Guideline for the Diagnosis and Treatment of Hypertrophic Cardiomyopathy: A report of the American College of Cardiology Foundation/American Heart Association Task Force on Practice Guidelines. Developed in collaboration with the American Association for Thoracic Surgery, American Society of Echocardiography, American Society of Nuclear Cardiology, Heart Failure Society of America, Heart Rhythm Society, Society for Cardiovascular Angiography and Interventions, and Society of Thoracic Surgeons. J. Am. Coll. Cardiol..

[37] Balaji S., DiLorenzo M.P., Fish F.A., Etheridge S.P., Aziz P.F., Russell M.W., Tisma S., Pflaumer A., Sreeram N., Kubus P. (2019). Risk factors for lethal arrhythmic events in children and adolescents with hypertrophic cardiomyopathy and an implantable defibrillator: An international multicenter study. Heart Rhythm..

[38] Pettersen M.D., Du W., Skeens M.E., Humes R.A. (2008). Regression equations for calculation of z scores of cardiac structures in a large cohort of healthy infants, children, and adolescents: An echocardiographic study. J. Am. Soc. Echocardiogr..

[39] Ommen S.R., Mital S., Burke M.A., Day S.M., Deswal A., Elliott P., Evanovich L.L., Hung J., Joglar J.A., Kantor P. (2020). 2020 AHA/ACC Guideline for the Diagnosis and Treatment of Patients With Hypertrophic Cardiomyopathy: A Report of the American College of Cardiology/American Heart Association Joint Committee on Clinical Practice Guidelines. Circulation.

[40] Semsarian C., French J., Trent R.J., Richmond D.R., Jeremy R.W. (1997). The natural history of left ventricular wall thickening in hypertrophic cardiomyopathy. Aust. N. Z. J. Med..

[41] Maron M.S., Olivotto I., Betocchi S., Casey S.A., Lesser J.R., Losi M.A., Cecchi F., Maron B.J. (2003). Effect of left ventricular outflow tract obstruction on clinical outcome in hypertrophic cardiomyopathy. N. Engl. J. Med..

[42] Geske J.B., Ong K.C., Siontis K.C., Hebl V.B., Ackerman M.J., Hodge D.O., Miller V.M., Nishimura R.A., Oh J.K., Schaff H.V. (2017). Women with hypertrophic cardiomyopathy have worse survival. Eur. Heart J..

[43] Javidgonbadi D., Andersson B., Abdon N.J., Schaufelberger M., Östman-Smith I. (2019). Factors influencing long-term heart failure mortality in patients with obstructive hypertrophic cardiomyopathy in Western Sweden: Probable dose-related protection from beta-blocker therapy. Open Heart.

[44] Lee C.H., Liu P.Y., Lin L.J., Chen J.H., Tsai L.M. (2007). Clinical characteristics and outcomes of hypertrophic cardiomyopathy in Taiwan--a tertiary center experience. Clin. Cardiol..

[45] El Saiedi S., Behairy N.H., Kharabish A., Esmail R., Seliem Z.S., Shafik M., El Mozy W. (2017). Delayed Myocardial Enhancement in Pediatric Hypertrophic Cardiomyopathy: Correlation with LV Function, Echocardiography, and Demographic Parameters. Pediatr. Cardiol..

[46] Marian A.J., Roberts R. (2001). The molecular genetic basis for hypertrophic cardiomyopathy. J. Mol. Cell. Cardiol..

[47] Nagueh S.F., McFalls J., Meyer D., Hill R., Zoghbi W.A., Tam J.W., Quinones M.A., Roberts R., Marian A.J. (2003). Tissue Doppler imaging predicts the development of hypertrophic cardiomyopathy in subjects with subclinical disease. Circulation.

[48] Maskatia S.A., Decker J.A., Spinner J.A., Kim J.J., Price J.F., Jefferies J.L., Dreyer W.J., Smith E.O., Rossano J.W., Denfield S.W. (2012). Restrictive physiology is associated with poor outcomes in children with hypertrophic cardiomyopathy. Pediatr. Cardiol..

[49] McMahon C.J., Nagueh S.F., Pignatelli R.H., Denfield S.W., Dreyer W.J., Price J.F., Clunie S., Bezold L.I., Hays A.L., Towbin J.A. (2004). Characterization of left ventricular diastolic function by tissue Doppler imaging and clinical status in children with hypertrophic cardiomyopathy. Circulation.

[50] Kaski J.P., Tome-Esteban M.T., Mead-Regan S., Pantazis A., Marek J., Deanfield J.E., McKenna W.J., Elliott P.M. (2008). B-type natriuretic peptide predicts disease severity in children with hypertrophic cardiomyopathy. Heart.

[51] Minami Y., Haruki S., Kanbayashi K., Maeda R., Itani R., Hagiwara N. (2018). B-type natriuretic peptide and risk of sudden death in patients with hypertrophic cardiomyopathy. Heart Rhythm..

[52] Ziółkowska L., Mazurkiewicz Ł., Petryka J., Kowalczyk-Domagała M., Boruc A., Bieganowska K., Ciara E., Piekutowska-Abramczuk D., Śpiewak M., Miśko J. (2021). The Indices of Cardiovascular Magnetic Resonance Derived Atrial Dynamics May Improve the Contemporary Risk Stratification Algorithms in Children with Hypertrophic Cardiomyopathy. J. Clin. Med..

[53] Ganame J., Mertens L., Eidem B.W., Claus P., D’Hooge J., Havemann L.M., McMahon C.J., Elayda M.A., Vaughn W.K., Towbin J.A. (2007). Regional myocardial deformation in children with hypertrophic cardiomyopathy: Morphological and clinical correlations. Eur. Heart J..

[54] McLeod C.J., Ackerman M.J., Nishimura R.A., Tajik A.J., Gersh B.J., Ommen S.R. (2009). Outcome of patients with hypertrophic cardiomyopathy and a normal electrocardiogram. J. Am. Coll. Cardiol..

[55] Mayosi B.M., Keavney B., Kardos A., Davies C.H., Ratcliffe P.J., Farrall M., Watkins H. (2002). Electrocardiographic measures of left ventricular hypertrophy show greater heritability than echocardiographic left ventricular mass. Eur. Heart J..

[56] Shah S., Nelson C.P., Gaunt T.R., van der Harst P., Barnes T., Braund P.S., Lawlor D.A., Casas J.P., Padmanabhan S., Drenos F. (2011). Four genetic loci influencing electrocardiographic indices of left ventricular hypertrophy. Circ. Cardiovasc. Genet..

[57] Haghjoo M., Mohammadzadeh S., Taherpour M., Faghfurian B., Fazelifar A.F., Alizadeh A., Rad M.A., Sadr-Ameli M.A. (2009). ST-segment depression as a risk factor in hypertrophic cardiomyopathy. Europace.

[58] Östman-Smith I., Wisten A., Nylander E., Bratt E.L., de-Wahl Granelli A., Oulhaj A., Ljungström E. (2010). Electrocardiographic amplitudes: A new risk factor for sudden death in hypertrophic cardiomyopathy. Eur. Heart J..

[59] Norrish G., Topriceanu C., Qu C., Field E., Walsh H., Ziółkowska L., Olivotto I., Passantino S., Favilli S., Anastasakis A. (2021). The role of the electrocardiographic phenotype in risk stratification for sudden cardiac death in childhood hypertrophic cardiomyopathy. Eur. J. Prev. Cardiol..

[60] Cortez D., Graw S., Mestroni L. (2016). In Hypertrophic Cardiomyopathy, the Spatial Peaks QRS-T Angle Identifies Those With Sustained Ventricular Arrhythmias. Clin. Cardiol..

[61] Gray B., Ingles J., Medi C., Semsarian C. (2013). Prolongation of the QTc interval predicts appropriate implantable cardioverter-defibrillator therapies in hypertrophic cardiomyopathy. JACC Heart Fail..

[62] Debonnaire P., Katsanos S., Joyce E., Van den Brink O.V., Atsma D.E., Schalij M.J., Bax J.J., Delgado V., Marsan N.A. (2015). QRS Fragmentation and QTc Duration Relate to Malignant Ventricular Tachyarrhythmias and Sudden Cardiac Death in Patients with Hypertrophic Cardiomyopathy. J. Cardiovasc. Electrophysiol..

[63] Molloy T.J., Okin P.M., Devereux R.B., Kligfield P. (1992). Electrocardiographic detection of left ventricular hypertrophy by the simple QRS voltage-duration product. J. Am. Coll. Cardiol..

[64] Walinder Osterberg A., Beausejour-Ladouceur V., Stephenson E.A., Mital S. (2021). Independent validation of the ECG Risk Score for Sudden Death Risk stratification in Pediatric Hypertrophic Cardiomyopathy. Circulation.

[65] Wålinder Österberg A., Östman-Smith I., Jablonowski R., Carlsson M.B., Green H., Gunnarsson C., Liuba P., Fernlund E. (2020). High ECG risk-scores predict late gadolinium enhancement on magnetic resonance imaging in HCM in the young. Pediatr. Cardiol..

[66] Östman-Smith I., Sjöberg G., Larsson P., Rydberg A., Fernlund E. (2021). Risk factors for sudden death in childhood—Differencies and similarities between non-syndrome associated hypertrophic cardiomyopathy and Noonan-group syndrome associated hypertrophic cardiomyopathy. Cardiol. Young..

[67] Kautzner J., Yi G., Camm A.J., Malik M. (1994). Short- and long-term reproducibility of QT, QTc, and QT dispersion measurement in healthy subjects. Pacing Clin. Electrophysiol..

[68] Crilley J.G., Boehm E.A., Blair E., Rajagopalan B., Blamire A.M., Styles P., McKenna W.J., Ostman-Smith I., Clarke K., Watkins H. (2003). Hypertrophic cardiomyopathy due to sarcomeric gene mutations is characterized by impaired energy metabolism irrespective of the degree of hypertrophy. J. Am. Coll. Cardiol..

[69] Raman B., Ariga R., Spartera M., Sivalokanathan S., Chan K., Dass S., Petersen S.E., Daniels M.J., Francis J., Smillie R. (2019). Progression of myocardial fibrosis in hypertrophic cardiomyopathy: Mechanisms and clinical implications. Eur. Heart J. Cardiovasc. Imaging.

[70] Jablonowski R., Fernlund E., Aletras A.H., Engblom H., Heiberg E., Liuba P., Arheden H., Carlsson M. (2015). Regional Stress-Induced Ischemia in Non-fibrotic Hypertrophied Myocardium in Young HCM Patients. Pediatr. Cardiol..

[71] Raphael C.E., Cooper R., Parker K.H., Collinson J., Vassiliou V., Pennell D.J., de Silva R., Hsu L.Y., Greve A.M., Nijjer S. (2016). Mechanisms of Myocardial Ischemia in Hypertrophic Cardiomyopathy: Insights from Wave Intensity Analysis and Magnetic Resonance. J. Am. Coll. Cardiol..

[72] Yetman A.T., McCrindle B.W., MacDonald C., Freedom R.M., Gow R. (1998). Myocardial bridging in children with hypertrophic cardiomyopathy—A risk factor for sudden death. N. Engl. J. Med..

[73] Biagini E., Pazzi C., Olivotto I., Musumeci B., Limongelli G., Boriani G., Pacileo G., Mastromarino V., Bacchi Reggiani M.L., Lorenzini M. (2016). Usefulness of Electrocardiographic Patterns at Presentation to Predict Long-term Risk of Cardiac Death in Patients With Hypertrophic Cardiomyopathy. Am. J. Cardiol..

[74] Michaelides A.P., Stamatopoulos I., Antoniades C., Anastasakis A., Kotsiopoulou C., Theopistou A., Misailidou M., Fourlas C., Elliott P.M., Stefanadis C. (2009). ST segment “hump” during exercise testing and the risk of sudden cardiac death in patients with hypertrophic cardiomyopathy. Ann. Noninvasive Electrocardiol..

[75] Spinner J.A., Noel C.V., Denfield S.W., Krishnamurthy R., Jeewa A., Dreyer W.J., Maskatia S.A. (2016). Association of Late Gadolinium Enhancement and Degree of Left Ventricular Hypertrophy Assessed on Cardiac Magnetic Resonance Imaging With Ventricular Tachycardia in Children With Hypertrophic Cardiomyopathy. Am. J. Cardiol..

[76] Axelsson Raja A., Farhad H., Valente A.M., Couce J.P., Jefferies J.L., Bundgaard H., Zahka K., Lever H., Murphy A.M., Ashley E. (2018). Prevalence and Progression of Late Gadolinium Enhancement in Children and Adolescents with Hypertrophic Cardiomyopathy. Circulation.

[77] Bonura E.D., Bos J.M., Abdelsalam M.A., Araoz P.A., Ommen S.R., Ackerman M.J., Geske J.B. (2020). Cardiac Magnetic Resonance Imaging Features in Hypertrophic Cardiomyopathy Diagnosed at <21 Years of Age. Am. J. Cardiol..

[78] Chaowu Y., Shihua Z., Jian L., Li L., Wei F. (2013). Cardiovascular magnetic resonance characteristics in children with hypertrophic cardiomyopathy. Circ. Heart Fail..

[79] Petryka-Mazurkiewicz J., Ziolkowska L., Kowalczyk-Domagala M., Mazurkiewicz L., Boruc A., Spiewak M., Misko J., Bieganowska K., Marczak M., Brzezinska-Rajszys G. (2020). LGE for Risk Stratification in Primary Prevention in Children with HCM. JACC Cardiovasc. Imaging.

[80] Norrish G., Qu C., Field E., Cervi E., Khraiche D., Klaassen S., Ojala T.H., Sinagra G., Yamazawa H., Marrone C. (2021). External validation of the HCM Risk-Kids model for predicting sudden cardiac death in childhood hypertrophic cardiomyopathy. Eur. J. Prev. Cardiol..

[81] Fernlund E., Sjöberg G., Alenius Dahlqvist J., Larsson P., Östman-Smith I. (2022). Assessment in a geographical cohort of PRIMaCY performance for risk stratification for sudden cardiac death in childhood hypertrophic cardiomyopathy. Cardiol. Young.

[82] McKenna W.J., Stewart J.T., Nihoyannopoulos P., McGinty F., Davies M.J. (1990). Hypertrophic cardiomyopathy without hypertrophy: Two families with myocardial disarray in the absence of increased myocardial mass. Br. Heart J..

[83] Varnava A., Baboonian C., Davison F., de Cruz L., Elliott P.M., Davies M.J., McKenna W.J. (1999). A new mutation of the cardiac troponin T gene causing familial hypertrophic cardiomyopathy without left ventricular hypertrophy. Heart.

[84] Coppini R., Ho C.Y., Ashley E., Day S., Ferrantini C., Girolami F., Tomberli B., Bardi S., Torricelli F., Cecchi F. (2014). Clinical phenotype and outcome of hypertrophic cardiomyopathy associated with thin-filament gene mutations. J. Am. Coll. Cardiol..

[85] Limongelli G., Pacileo G., Calabrò R. (2006). Is sudden cardiac death predictable in LEOPARD syndrome?. Cardiol. Young..

[86] Eichhorn C., Voges I., Daubeney P.E.F. (2019). Out-of-hospital cardiac arrest and survival in a patient with Noonan syndrome and multiple lentigines: A case report. J. Med. Case Rep..

[87] Jorholt J., Formicheva Y., Vershinina T., Kiselev A., Muravyev A., Demchenko E., Fedotov P., Zlotina A., Rygkov A., Vasichkina E. (2020). Two New Cases of Hypertrophic Cardiomyopathy and Skeletal Muscle Features Associated with ALPK3 Homozygous and Compound Heterozygous Variants. Genes.

[88] Wolf C.M., Zenker M., Burkitt-Wright E., Edouard T., García-Miñaúr S., Lebl J., Shaikh G., Tartaglia M., Verloes A., Östman-Smith I. (2022). Management of cardiac aspects in children with Noonan syndrome—Results from a European clinical practice survey among paediatric cardiologists. Eur. J. Med. Genet..

[89] Sherrid M.V. (2013). Implantable cardioverter-defibrillators for children and adolescents at high risk for sudden death from hypertrophic cardiomyopathy. J. Am. Coll. Cardiol..

[90] Christiaans I., van Engelen K., van Langen I.M., Birnie E., Bonsel G.J., Elliott P.M., Wilde A.A. (2010). Risk stratification for sudden cardiac death in hypertrophic cardiomyopathy: Systematic review of clinical risk markers. Europace.

